# Self-Assembled Polyaniline/Ti_3_C_2_T_x_ Nanocomposites for High-Performance Electrochromic Films

**DOI:** 10.3390/nano11112956

**Published:** 2021-11-04

**Authors:** Tao Lin, Wenlong Liu, Bin Yan, Jing Li, Yi Lin, Yinghui Zhao, Zheng Shi, Sheng Chen

**Affiliations:** 1 School of Mechanical Engineering, Chengdu University, Chengdu 610106, China; lintao@stu.cdu.edu.cn; 2 College of Biomass Science and Engineering, Sichuan University, Chengdu 610065, China; yanbinscu@126.com (B.Y.); linasdn@126.com (Y.L.); yh18428367250@163.com (Y.Z.); drshiz1002@hotmail.com (Z.S.); 3 Institute for Advanced Materials Deformation and Damage from Multi-Scale, Chengdu University, Chengdu 610106, China; 4 College of Food and Biological Engineering, Chengdu University, Chengdu 610106, China; lijing@cdu.edu.cn

**Keywords:** self-assembly, MXene, polyaniline, nanostructures, electrochromic electrode

## Abstract

Electrochromic materials and devices are attracting intense attention because of their low energy consumption and open-circuit memory effect. Considering the difficult processing characteristics of electrochromic conductive polymers, we developed a facile and scalable strategy to prepare solution processable polyaniline (PANI)-based nanocomposites by introducing two-dimensional titanium carbon nanosheets (MXene) through a self-assembly approach. The PANI/MXene nanocomposite can be fabricated into porous films via spray-coating process, which show an obvious synergetic effect of both materials, leading to superior electrochromic properties. The optical contrast of the optimized PANI/MXene film reached as high as 55% at =700 nm, and its response times were 1.3 s for coloration and 2.0 s for bleaching, respectively. In addition, the composite film also showed excellent cycle stability (after 500 cycles, the ΔT retention was above 87%). The improved electrochromic properties are owed to the high conductivity of MXene and the formation of the porous composite film structure, which promote the electronic/ionic transfer and migration efficiency. This research suggests that the self-assembly method and the conductive polymer/MXene nanocomposites have a potential application in the fields of electronic functional films and devices.

## 1. Introduction

The development of industrial society has been accompanied by energy problems and environmental pollution, which have become serious problems that mankind must face [1]. Nearly 40% of the total social energy consumption in buildings has become the primary target for energy efficiency [2,3]. Electrochromic (EC) smart windows with reversible optical changes in visible light and near infrared bands by applying different potentials are promising for using as energy-saving windows [4]. In addition to energy-saving windows, electrochromic materials and devices can also be used as low-power displays, adaptive camouflage apparatuses, coloration-changing sunglasses, anti-glare rearview mirrors, and so on [5,6,7].

Electrochromic materials are mainly divided into transition metal oxides, organic compounds, and polymers [8,9,10]. The transition metal oxides, such as tungsten oxide and nickel oxide, have been widely used due to their high transmittance contrast (ΔT%) and stability. However, many of these materials are normally fabricated into films using the conventional magnetron sputtering method, which is expensive and inefficient because of the special equipment and high vacuum conditions [11]. More research is required for a facile strategy to reach the balance between electrochromic performance and a low-cost film fabrication method. Conductive polymers have attracted more and more attention because of their diverse process methods, such as cast coating, electrochemical deposition, spin coating, ink-jet printing, magnetron sputtering, and others [12,13,14,15]. Among the conductive polymers, polyaniline (PANI) is considered to be one of the most suitable candidates for electrochromic application due to the obvious coloration contrast and fast switching ability between its redox states. PANI also has the advantages of low-cost, excellent electrochemical stability, and good environmental stability [16]. Recently, lots of reports have focused on further improving the electrochromic performances of PANI using different strategies. Inamdar et al. [17] successfully prepared mesoporous Ni-PANI thin film electrodes on indium-doped tin oxide (ITO) conductive glass substrates by pulsed electro-polymerization and obtained an excellent optical modulation of 55.6%. Zhang et al. [18] prepared urchin-like WO_3_/PANI nanobelts by solvothermal and electropolymerization methods, which showed a distinct optical modulation (ΔT about 45%) and excellent durability after long-term cycles. Therefore, the combination of PANI and inorganic nanomaterials can improve the electrochromic properties of PANI, but the preparations of the above-mentioned nanocomposite films usually require harsh conditions and tedious synthesis process, or adopting the electrochemical deposition method, which limit their large-scale production and practical application.

MXene is a general term for new two-dimensional transition metal carbides or nitrides, of which the representative ones are Ti_3_C_2_T_x_ (T = F, OH, etc.). The high electrical conductivity, large specific surface area, and excellent electrochemical activity exhibited by MXene provide opportunities to combine and enhance the electrical activity of conducting polymers in batteries, supercapacitors, and electromagnetic interference shielding [19,20,21,22]. Li et al. [23] developed a thin and optically transparent micro-supercapacitor using MXene-Poly (3,4-ethylenedioxythiophene) (PEDOT) hybrid films by electrochemical deposition. Though only a slight electrochromic behavior was observed, they found that MXene could provide high electronic conductivity while the MXene-PEDOT composites were used as energy-storage materials. Another recent work revealed that MXene effectively enhanced the transportation of ions and electrons both when it was mixed with inorganic electrochromic materials [24]. The above studies showed that MXene is suitable for use as the auxiliary material of electrochromic films.

**Figure 1:**
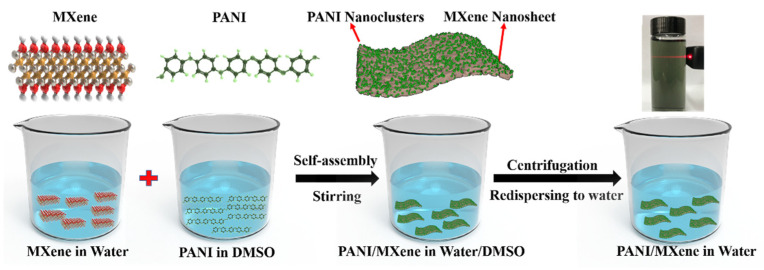
Schematic of the synthesis of PANI/MXene composites.

In this paper, we designed a nanocomposite of PANI and MXene, which combines their electrochromic properties and electronic conductivity. This design was not only obtaining a PANI/MXene nanocomposite with good electrochromic performances, but it also developed a facile and scalable strategy to fabric the composite. We prepared the water-dispersible PANI/MXene composite through a simple self-assembly technique (Figure 1). The obtained PANI/MXene suspensions were spray-coated onto transparent conductive substrate (ITO glass), forming a homogeneous and porous film in which, the PANI nanoparticle-decorated MXene overlapped with each other, facilitating the transportation of ions and electrons both. As a result, the PANI/MXene film demonstrated excellent performance in optical modulation (ΔT above 55% at 700 nm) and cyclic stability (ΔT retention rate > 87% after 500 cycles). In addition, the electrochromic electrode also showed fast response times (1.3 s for coloration, and 2.0 s for bleaching). Based on the spray-coating film-formation technology, we fabricated a patterned electrochromic film, illustrating the potential of the PANI/MXene hybrid material in electrochromic labels or displays.

## 2. Materials and Methods

### 2.1. Materials

Bulk Ti_3_AlC_2_ (CAS-No: 196506-01-1) powder was purchased from 11 Technologies Co. Ltd., Changchun, China and hydrochloric acid (HCI, CAS-No: 7647-01-0) (37 w%) and lithium fluorides (LiF, CAS-No: 7789-24-4) were supplied by Sigma-Aldrich (Sigma-Aldrich, Inc., St-Louis, MO, USA). Aniline (CAS-No: 62-53-3) and ammonium persulfate (CAS-No: 7727-54-0) were provided by Aladdin (Shanghai, China). Aqueous ammonia (CAS-No: 1336-21-6) and ethanol (CAS-No: 64-17-5) were purchased from Kelong Chemical Reagent Co, Ltd. (Chengdu, China). All chemicals used throughout the experiments were of analytical grade, and the water used for the experiments was deionized water (CAS-No: 7732-18-5). Indium tin oxide (ITO) conductive glass (sheet resistance, 8.0 Ω/sq; dimensions, 30 mm × 20 mm; thickness, 1 mm) was supplied by Shenzhen South China Xiang Cheng Science and Technology Co., Ltd. (Shenzhen, China).

### 2.2. Methods

#### 2.2.1. Preparation of Ti_3_C_2_T_x_ MXene

Ti_3_C_2_T_x_ was prepared by etching of aluminum from Ti_3_AlC_2_, and the MILD method was used to induce delamination during synthesis [25]. Firstly, 2 g LiF was dissolved in a Teflon beaker including 40 mL of 12 M HCl under magnetic stirring. Then, 2 g Ti_3_AlC_2_ powder was slowly added into above solution. After etching at 40 °C under ventilated conditions for 24 h, the Ti_3_C_2_T_x_ slurry was centrifuged and washed repeatedly at 5000 rpm until the pH was about 6. When the pH was ≥6, a dark green suspension containing bulk Ti_3_C_2_T_x_ MXene was formed. At this time, the above suspension was transferred into a breaker containing 100 mL deionized water, which were further sonicated in ice water for 30 min to obtain the exfoliated Ti_3_C_2_T_x_ MXene. Then, a stable dark green exfoliated MXene solution was collected by centrifugation of above solution at 3500 rpm. The solid content of the aqueous dispersion was measured by differential weight method. Then, a calculated amount of deionized water was added to adjust the solid content of the dispersion to 10 mg/mL.

#### 2.2.2. Synthesis of PANI

The PANI used in the experiment was synthesized by the conventional chemical oxidation polymerization method. Approximately 4.66 g of aniline was dispersed in 25 mL of 1 M HCl solution, and the pH was adjusted to 1.0 with an acid solution. Subsequently, 14.26 g of ammonium persulfate was dissolved in 25 mL deionized water and added into the reaction solution at 0 °C. After 2 h, the mixture was filtrated and repeatedly washed with deionized water. After that, the product was put into ammonia solution and stirred for another 24 h to obtain dedoping PANI, then filtered and washed by deionized water and ethanol, respectively. Finally, the filtered solid was dried under vacuum at 80 °C for 12 h to obtain dark blue powdered PANI.

#### 2.2.3. Preparation of PANI/MXene Nanocomposite and Electrochromic Films

The PANI/MXene composite was synthesized by self-assembly of PANI nanoparticles and MXene nanosheets. First, 0.5 g of ground PANI powder and 50 mL of DMSO were mixed and stirred 4 h at room temperature. Then different amounts of PANI/DMSO solution were slowly added into the previously obtained MXene aqueous suspension under stirring. The PANI/MXene floccule gradually formed the mixture. PANI/MXene composites with different mass ratios (the film samples of 9PANI/1MXene, 7PANI/3 MXene, 5PANI/5MXene, 3PANI/7Mxene, and 1PANI/9Mxene fabricated using the nanocomposites with different mass ratios of PANI and MXene of 9:1, 7:3, 5:5, 3:7, and 1:9, respectively) were thus obtained by centrifugation and repeatedly washed with water for the removal of DMSO solvent. Then the collected PANI/MXene composites were doped with a few drops of 1 M HCl and redispersed in an aqueous solution to obtain an PANI/MXene suspension. For comparison, the Pure PANI particles was prepared through the same procedure without the addition of MXene. Before the fabrication of the electrochromic films, the ITO glassed with the size 20 mm × 30 mm × 1 mm were washed with petroleum ether and acetone to remove contaminants, and then washed continuously with ethanol and deionized water for 15 min, and then air-dried. Pure PANI, MXene, and PANI/MXene nanocomposite films were prepared by spraying corresponding suspension onto the washed ITO glass substrates on a hot plate at 100 °C.

### 2.3. Characterization

The PANI/MXene films were structurally analyzed by X-ray diffraction (XRD, TD-3500, Dandong Tongda Science & Technology Co., Ltd., Dandong, China) with voltage and current set to 40 kV and 44 mA, respectively. The step scan was 0.03° and 2θ range was 5°–70°. The morphologies of the samples were characterized by field-emission scanning electron microscopy (FE-SEM, JEOLJSM-5900LV, JEOL, Ltd., Tokyo, Japan) and transmission electron microscopy (TEM, G2-F20, JEOL, Ltd., Tokyo, Japan). The optical properties of the PANI/MXene films were examined by UV-vis spectroscopy (UV3600, Beijing Purkinje General Instrument Co., Ltd., Beijing, China), coupled with the Electrochemical Workstation (CHI 660, Shanghai Chenhua Instrument Co., Ltd., Shanghai, China) to apply electrochemical potentials. Surface chemical compositions were analyzed by X-ray photoelectron spectroscopy (XPS, AXIS Ultra DLD, Kratos Analytical, Manchester, UK). Fourier infrared (FT-IR) spectra of the samples were obtained by FT-IR spectroscopy (FT-IR, Nicolet 6700, Thermo Fisher Scientific, Waltham, MA, USA).

### 2.4. Electrochemical Measurements

The electrochemical performance of the PANI/MXene composite film was tested in a three-electrode system, and the electrolyte was 0.1 M HCl solution. In the three-electrode system, the ITO glass coated with PANI/MXene composites was served as the working electrode (20 mm × 15 mm), the saturated calomel electrode was served as the reference electrode, and the platinum electrode (10 mm × 10 mm) was served as the counter electrode. Cyclic voltammetry (CV) was employed to measure their electrochemical activity. The Cyclic Voltammetry (CV) properties of the PANI/MXene electrochromic film were determined using a CHI 660 electrochemical workstation (Shanghai Chenhua Instruments Co. Ltd., Shanghai, China) with the voltage range from −0.4 to +1.0 V and scan rate ranged from 10 to 100 mV/s; 0.1 M HCl aqueous solution was used as the electrolyte solution.

The electrochemical impedance spectroscopy (EIS) was carried out at the open circuit potential with an amplitude of 5 mV in a frequency range from 0.01 Hz to 100 kHz. The electrolyte solution was the same with the CV test. The spectroelectrochemical curves were measured using a TU-1900 UV-vis spectrophotometer (Beijing Purkinje General Instrument Co., Ltd., Beijing, China) coupled with the Electrochemical Workstation for the application of electrochemical potential.

## 3. Results and Discussion

The synthesis schematic diagram of Ti_3_C_2_T_x_ MXene and PANI/MXene composite materials is shown in Figure 1. First, in situ HF was formed by LiF and HCl to etch Al in Ti_3_AlC_2_ to obtain multilayer MXene. During the etching process, end groups T containing oxygen (-O), hydroxyl (-OH) and/or fluorine (-F) groups were introduced into Ti_3_C_2_T_x_ MXene. Subsequently, a few layers of Ti_3_C_2_T_x_ MXene nanosheets were obtained through vigorous manual shaking and ultrasonic treatment. Because of the existence of hydrophilic end groups, the prepared few-layer MXene nanosheets were easy to disperse in water and form Tyndall phenomenon (Figure S1). PANI was prepared by conventional chemical oxidative polymerization, followed by dissolving in the DMSO solvent. PANI/MXene composite was synthesized by the self-assembly method. Firstly, the DMSO solution of PANI was dropped into the MXene suspension to form a PANI/MXene composite precipitate. Second, composite sediments were concentrated by washing with water repeatedly to remove the DMSO solvent. The obtained PANI/MXene composites can be well redispersed in water because of the hydrophilic end groups of PANI/MXene composites (Figure 1).

**Figure 2:**
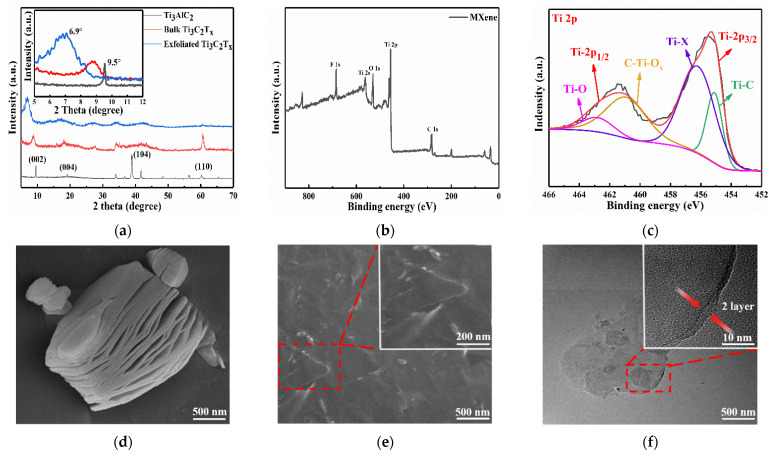
(**a**) XRD patterns of Ti_3_AlC_2_, bulk Ti_3_C_2_T_x_ MXene, and exfoliated Ti_3_C_2_T_x_ MXene. The inset shows magnified spectrum 5° to 12°; (**b**) XPS spectrum of full scan of exfoliated Ti_3_C_2_T_x_ MXene; (**c**) high-resolution Ti 2p XPS spectra of exfoliated Ti_3_C_2_T_x_ MXene.; (**d**) SEM image of bulk Ti_3_C_2_T_x_ MXene; (**e**) SEM images with different degrees of magnifications of exfoliated Ti_3_C_2_T_x_ MXene; (**f**) HRTEM images of the exfoliated Ti_3_C_2_T_x_ with different magnifications.

The physical and chemical structures of the delaminated MXene as shown in Figure 2a–c were analyzed by X-ray diffraction (XRD) and X-ray photoelectron spectroscopy (XPS). In Figure 2a, the exfoliated Ti_3_C_2_T_x_ showed an obvious (002) characteristic peak at 2θ = 6.9°, which is smaller than that of the Ti_3_AlC_2_ (2θ = 9.5°) [26]. It is owed to the removal of the Al layer in Ti_3_AlC_2_ during etching process and the intercalation effect of lithium ions. The delaminated Ti_3_C_2_T_x_ sheets were separated after hand-shaking and sonication. The layer spacing increased further, corresponding to an increase in d-spacing from 0.98 nm to 1.30 nm. We found the disappearance of the strongest (104) diffraction peak both in the XRD curves of bulk Ti_3_C_2_T_x_ and exfoliated Ti_3_C_2_T_x_ while the (002) and (004) diffraction peaks were weakened (Figure 2a). This indicated that the Al in Ti_3_AlC_2_ was completely removed, which is consistent with the reported research [25,27]. The surface chemical structure of MXene was characterized by XPS in Figure 2b. The results showed that MXene is mainly composed of C, O, Ti, and F. In Figure 2c, Ti 2p is equipped with two pairs of dipole moments (Ti 2p_3/2_-Ti 2p_1/2_), which is consistent with Martin’s report [28]. The main peaks of Ti 2p_3/2_ were concentrated at 455.1 and 456.2 eV, and the diffraction peak at 455.1 eV corresponds to Ti^2+^, indicating the existence of the Ti–C bond; meanwhile, the diffraction peak at 456.2 eV corresponds to the Ti–X binding site [29]. The main peaks of Ti 2p_1/2_ were concentrated at 460.9 and 462.8 eV; the diffraction peak at 460.9 eV corresponds to C–Ti–O_x_; and that at 462.8 eV corresponds to the presence of the Ti–O bond.

The formation of the Ti_3_C_2_T_x_ MXene nanosheets was observed by the SEM and TEM images. As shown in Figure 2d, after etching by using the MILD method [25], the bulk Ti_3_C_2_T_x_ exhibited an accordion shape, which is attributed to the removal of the Al layer and hydrogen precipitation. This observation indicates a strong interaction between the etched Ti_3_C_2_T_x_ layers. After further shaking and ultrasonic treatment, the exfoliated MXene nanosheets with smooth surfaces were observed in the SEM images of Figure 2e. Meanwhile, the obvious wrinkle structure can be observed from Figure S2, which illustrates the flexibility of the exfoliated few layer Ti_3_C_2_T_x_ MXene. To further understand the morphology of MXene nanosheets, the TEM test was performed (Figure 2f). It was also found that the MXene nanosheet exhibited layered characteristics. As shown in the inset of Figure 2f, the number of the layers was about two. Therefore, the results suggested that few-layer MXene sheets were successfully prepared.

**Figure 3:**
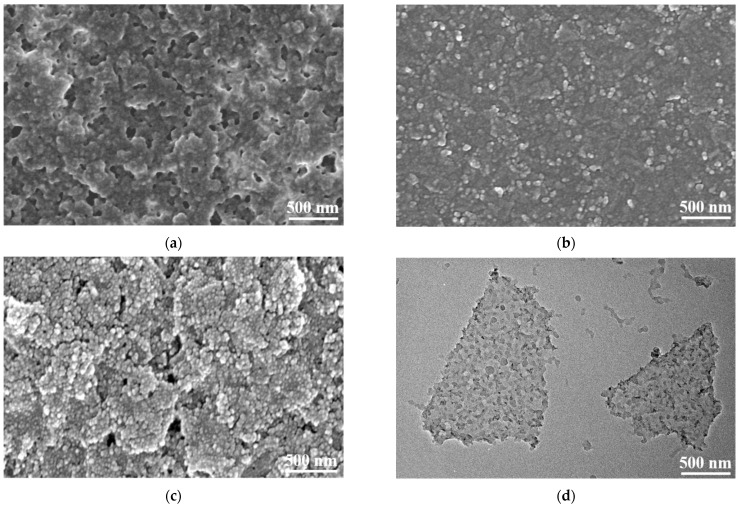
(**a**) SEM images of the spray-coated MXene film; (**b**) The SEM images of PAN film; (**c**) SEM images of the spray-coated PANI/MXene film; (**d**) TEM morphology of the PANI/MXene film.

Inspired by the nanoprecipitation method [30], the PANI/MXene nanocomposites were synthesized by the self-assembly of PANI nanoclusters and MXene nanosheets. As shown in Figure 3, MXene, PANI, and PANI/MXene thin films can be readily fabricated on ITO substrates by spray-coating. The exposed MXene nanosheet showed irregular structures, which overlapped with each other and formed holes or channels (Figure 3a). In addition, the intrinsic high conductivity and porous structure of the film render it suitable as a component for electrochromic materials. However, as shown in Figure 3b and Figure S3, the film prepared by spray-coating the pure PANI nanoclusters presented a dense structure, which is more compact than that of the MXene film. For the electrochromic film, this dense structure could further hinder the diffusion of electrolyte ions at the film–electrolyte interface [31]. During the electrochemical process, the interconnecting gap around each MXene increased the surface area and provided a sufficient area for the accumulation and diffusion of electrolyte ions. Therefore, the monolithic porous structure of PANI/MXene composites formed by assembling PANI particles on MXene substrates can potentially improve the electrochromic performance of PANI. The microstructure and morphology of PANI/MXene films are exhibited in Figure 3c. The results showed that PANI nanoclusters are decorated on MXene nanosheets, forming a rough surface structure, which can effectively resist the aggregating and compacting of the PANI nanoclusters. At the edge of the PANI/MXene film, as shown in Figure S4a, we can see clearly that all the PANI nanoparticles were uniformly assembled on the surface of MXene nanosheets, while no PANI particles were coupled with MXene, indicating a strong interaction between PANI and MXene during the self-assembly process (Figure S4a).

The good combination of PANI and MXene is attributed to the hydrogen bond interaction between the N-H group of PANI macromolecules and the terminated O on the surface of MXene nanosheets during the self-assembly process [32]. The rough surface of the PANI/MXene composite material with high specific surface area also exposes more active sites for contact with electrolyte ions, which can promote the ion doping rate of the electrode. The PANI/MXene composites were further measured by the TEM tests as shown in Figure 3d and Figure S4b. The images exhibited that PANI nanoclusters were uniformly distributed on the surface of MXene nanosheets, and the composites had good dispersibility. It is worth noting that the PANI/MXene composites also exhibited foldable features, which further illustrates the flexibility of the few-layered MXene (Figure S4c). The few-layers structure and high specific surface area of the as prepared MXene also play an important role in the formation of the nanocomposites.

**Figure 4:**
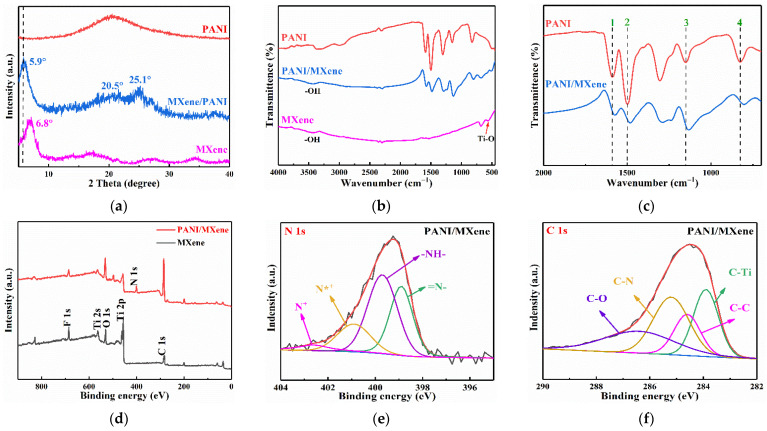
(**a**) XRD patterns of MXene, PANI and PANI/MXene; (**b**) FT–IR spectra of MXene, PANI, and PANI/MXene; (**c**) FT–IR spectra of PANI/MXene with a wavenumber at 2000 to 700 cm^−1^. Lines 1 and 2 represent the redshifts of the absorption bands of quinone ring and the benzene ring, respectively. Lines 3 and 4 represent the redshifts of the C-H bending vibration absorption bands. (**d**) XPS analysis of pure MXene and PANI/MXene; (**e**) N 1s and (**f**) C 1s high-resolution spectra scans of PANI/MXene.

Figure 4a presents the XRD patterns of MXene, PANI, and PANI/MXene. The characteristic peaks at 6.9°, 16.8°, 27.2°, and 34.2° correspond to the (002), (004), (008), and (0010) reflections of MXene, respectively [33]. The spectrum of pure PANI shows a single, broad diffraction peak between 15° and 30°, which is the characteristic peak of the amorphous emeraldine form of PANI [34]. The spectrum of PANI/MXene composites exhibited the characteristic peaks of both PANI and MXene. The two diffraction peaks at 20.5° and 25.1° are ascribed to the (020) and (200) periodic reflection planes parallel and perpendicular to the PANI chains, respectively [35]. Compared with the diffraction spectrum of pure PANI, these two peaks were sharper, indicating a relatively ordered structure of the PANI chain in the PANI/MXene composites. This ordered structure results from higher crystallinity, which is conducive to the improvement of the electrical conductivity of PANI. Notably, the (002) peak of PANI/MXene decreased to 5.9° relative to MXene (6.9°), corresponding to the increase in interplanar spacing (d-spacing) from 10.1 Å to 13.2 Å. This significant increase in d spacing may be attributed to the intercalation of DMSO into MXene interlayers during the preparation process of composites. Figure 4b,c present the FT-IR spectra of MXene, PANI, and PANI/MXene. The characteristic peaks of MXene at 3415 cm^−1^ are attributed to the interlayer adsorption of water molecules [36]. The characteristic peak at 572 cm^−1^ could be attributed to Ti-O [37]. The C=C stretching vibrations of the quinone and benzene rings correspond to the characteristic peaks of PANI at 1587 and 1498 cm^−1^, respectively [38]. For PANI/MXene, there were characteristic peaks of PANI and MXene presented in both (Figure 4b), indicating that the self-assembly was successful. The characteristic peak at 817 cm^−1^ corresponds to the bending vibration of the C-H plane of the aromatic ring of PANI, while the characteristic peak at 1147 cm^−1^ corresponds to the bending vibration of the C-H [39]. However, as shown in Figure 4c, for the PANI/MXene nanocomposite, the absorption bands of quinone ring and the benzene ring at 1587 cm^−1^, and 1498 cm^−1^ red shifted to 1574 cm^−1^, and 1481 cm^−1^, respectively. as Additionally, the C-H bending vibration absorption bands at 1149 cm^−1^ and 825 cm^−1^ red shifted to 1134 cm^−1^ and 804 cm^−1^ respectively. The shift may be attributed to the formation of hydrogen bonding between MXene sheets and the imine group of PANI [37].

The chemical states and elemental compositions of MXene and PANI/MXene were further examined by XPS (Figure 4d–f and Figure S5). The XPS survey spectra of these two materials exhibited the presence of peaks corresponding to Ti, C, O, N, and F. High-resolution N 1s spectrum of the PANI/MXene revealed four characteristic nitrogen-moiety peaks indicating different structures of PANI (Figure 4e). The peak with binding energy of 398.9 eV corresponds to the quinone imine (=N-) structure, while the peak at 399.7 eV corresponds to the benzene amine (-NH-) [40]. The peaks at 400.9 eV and 402.6 eV correspond to the nitrogen atom with a delocalized positive charge N*^+^ and the protonated amine unit, respectively [41]. The degree of oxidation and protonation of PANI can be determined by analyzing the ratio of =N-, -NH-, and cationic nitrogen [42]. As summarized in Table S1, the PANI in PANI/MXene contained ~32.68% imine nitrogen and ~45.38% amine nitrogen. The ratio of =N- to -NH- was 0.72, which was caused by the doping process of HCI. The high-resolution C 1s spectrum of PANI/MXene (Figure 4f) exhibited four characteristic peaks at 283.9 (C-Ti), 284.6 (C-C), 285.2 (C-N), and 286.4 eV (C-O), respectively [43]. The Ti 2p spectrum for PANI/MXene is shown in Figure S5. Note that the electronic properties of PANI determine the conductivity of PANI. The process of HCl doping leads to the protonation of PANI nitrogen atoms and further to the delocalization of charge carriers on the framework.

**Figure 5:**
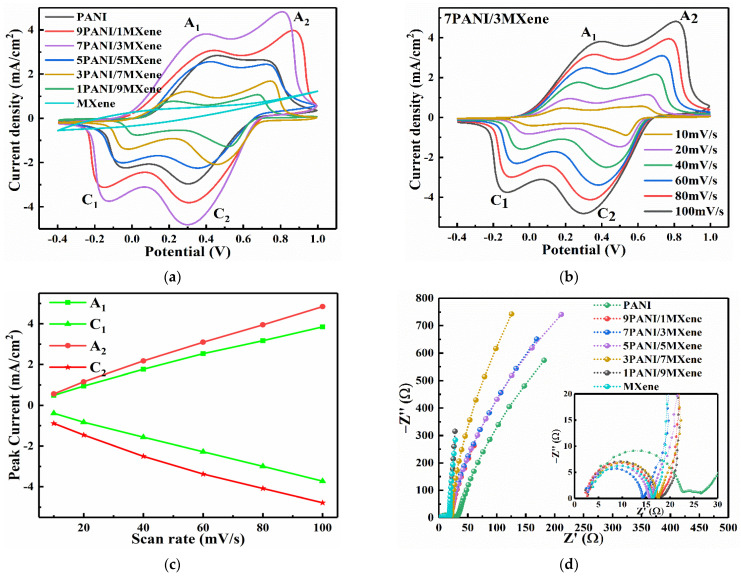
(**a**) CV curves of PANI/MXene hybrids electrodes with different mass ratios at the scan rate of 100 mV/s; (**b**) CV curves of the 7PANI/3PANI hybrids electrode at different scan rates; (**c**) Relationships of peak current density vs. potential scan rate; (**d**) Nyquist plots of PANI/MXene hybrids electrodes with different mass ratios in 0.1 M HCl in the frequency range of 10^−2^–10^5^ Hz.

MXene, PANI, and PANI/MXene films were fabricated by spraying the aqueous suspension on the surface of ITO glasses. We used a three-electrode system to characterize the electrochemical performance of the films in a 0.1M HCl electrolyte. In Figure 5a, all CV curves have two distinct pairs of redox peaks except for the pure MXene, which originate from the redox transition of PANI between the fully reduced leucoemeraldine base (LB) form, the half oxidized emeraldine base (EB) form, and the fully oxidized pernigraniline base (PB) form [44]. The first peak in all curves may be caused by the formation of p-benzoquinone and hydroquinone, and oxidation at +0.81 V related to the formation of a PANI polymerizationchain [45]. The serious agglomeration of the pure PANI nanoparticles led to the reduction of effective electrochemical active specific surface area and the limitation of peak current density. In our past work, we found that Pt(E)/Pt(S)/PCP electrodes with higher surface area exhibited higher redox currents than Pt(S)/PCP electrodes with relatively compact surface areas [46]. Compared with the pure PANI electrode, the 7PANI/3MXene electrode showed larger redox charge capacities and higher peak current density, indicating the excellent synergistic effect of MXene and PANI. The CV performance of 7PANI/3MXene electrode at different scan rates is shown in Figure 5b. When the scan rate was at 100 mV s^−1^, the first pair of redox peak positions A_1_/C_1_ were located at +0.37 V/−0.13 V. It is attributed to the redox process between colorless LB form PANI (light yellow) and slightly darker emeraldine salt (ES) form (green) with the p-doping and de-doping of the polymer chains. The second pair of redox peaks A_2_/C_2_ were located at +0.81 V/0.30 V, corresponding to the conversion of EB form (green) and pernigraniline salt (PS) form (dark blue), which is also the p-doping/de-doping process [47]. As shown in Figure 5b, we can also see that with the scan rate applied from 10 to 100 mV/s, anodic peak potentials were shifted toward more positive potential values while cathodic peak potentials were shifted to more negative potential values [48]. The CV curves of 7PANI/3MXene film maintained a stable form at different scanning rates even as high as 100 mV s^−1^, indicating the excellent doping and de-doping rates, which may be related to the porous structure and good conductivity introduced by the addition of MXene. Figure 5c showed the peak current densities for the oxidation and reduction peaks. The current densities of the PANI/MXene electrode are linearly dependent on the scan rates over the range of 10–100 mV/s. Such linear relationships of the currents are the manifestation of a surface-controlled reaction [49]. Therefore, it can be concluded that the redox reaction of the PANI/MXene electrode is limited by the ion diffusion from the electrolyte solution to the electrode surface [44].

Electrochemical impedance spectroscopy (EIS) is an effective method for studying electrical conductivity and chemical transformations in electrochemical processes [50]. To investigate the enhancement of the electrochemical performance of PANI/MXene electrodes by MXene nanosheets, Figure 5d shows the EIS profiles of PANI/MXene with different mass ratios. The Nyquist plots of all electrodes contained a semicircle in the high frequency region and a straight line in the low frequency region, indicating that the electrochemical processes at PANI/MXene electrodes are jointly controlled by charge transfer and ion diffusion. The relatively small radius of the high frequency region of the Nyquist diagram indicates a low charge transfer resistance. Thus, as shown in Figure 5d, 7PANI/3MXene had the lowest charge transfer resistance between the composite film and the ITO conductive layer, and between the stacked nanosheets, which can improve the electrochemical performance [51,52]. In addition, the electrodes with different MXene contents had different slopes of the straight lines in the low frequency region. Compared with the pure PANI electrode, 7PANI/3MXene had a steep slope in the low frequency region, indicating that the composite electrode has higher ion conductivity and shorter ion diffusion path [53]. In order to quantify the different parameters of the PANI/MXene electrode EIS test, we fit the EIS data into an equivalent circuit, as shown in (Figure S6). In the fitting circuit parameters, Rs, Rct, and W correspond to solution resistance, charge transfer resistance, and Warburg resistance, respectively [54,55]. The fitting results showed that the equivalent circuit is suitable for the characterization of the PANI/MXene electrochemical system. The composite electrode showed lower Rs and Rct values (Table S2), indicating that the 7PANI/3MXene electrode improved the charge/ion transfer rate [55]. The lower W value of the 7PANI/3MXene electrode compared with the pure PANI also indicates a faster ion diffusion rate of the composite electrode. This may be related to the larger specific surface area and higher porosity of the composite electrode. These findings are consistent with the CV measurement results.

**Figure 6:**
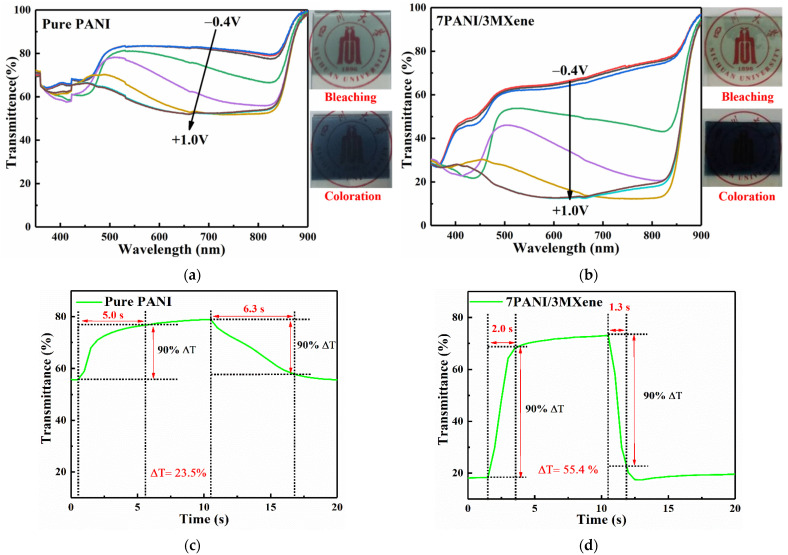
(**a**,**b**) UV—vis transmittance spectra of Pure PANI and PANI/MXene films performed at different applied potentials. The curves of different colors correspond to the UV—vis transmittance spectra of the films under different constant voltages (−0.4 to +1.0 V). (**c**,**d**) In situ optical transmittance responses of PANI and PANI/MXene with applied potentials of −0.4 and +1.0 V for 10 s per step measured at 700 nm.

The electrochromic performance is typically evaluated by optical contrast (ΔT), response time (τ), and cycling stability [56]. The spectroelectrochemical curves of pure PANI and PANI/MXene films are presented in Figure 6a,b at different potentials (the spectra of the other samples are shown in Figure S7a–d). The transmittances of the films markedly decreased when the applied voltages changed from −0.4 to 1.0 V. Thus, PANI-based film are anodic-coloration electrochromic materials at the positive potentials. The ΔT values were expressed by the difference of the transmittances where the color contrast between the coloration state and the bleaching state was the largest (for the pure PANI electrode, it was at about 700 nm). The ΔT of pure PANI film was determined to be 23.5%, while that of the PANI/MXene film was 55.4%, which is 31.9% higher than that of the pure PANI film. It shows that the optical contrast of the PANI/MXene nanocomposite film at visible light wavelength has a great improvement.

Response time is an important indicator to measure the performance of electrochromic materials. It is defined as the time it takes for the material to reach 90% of the complete color change during the transition between the coloration and the bleaching state. Figure 6c,d are the transmittance-time plots of PANI and PANI/MXene films at 700 nm, respectively. It can be seen that the bleaching time (τ_b_) and coloration time (τ_c_) of the PANI/MXene composite films were 2.0 s and 1.3 s, respectively, which are obviously lower than those of the pure PANI film (τ_b_ is 5.0 s, and the τ_c_ is 6.3 s). Therefore, the PANI/MXene film has faster response speeds. It may be due to the lower charge transfer resistance and faster ion diffusion rate of the nanocomposite film, which is conducive to the electrochemical reaction and penetration or diffusion of ions inside the film.

**Figure 7:**
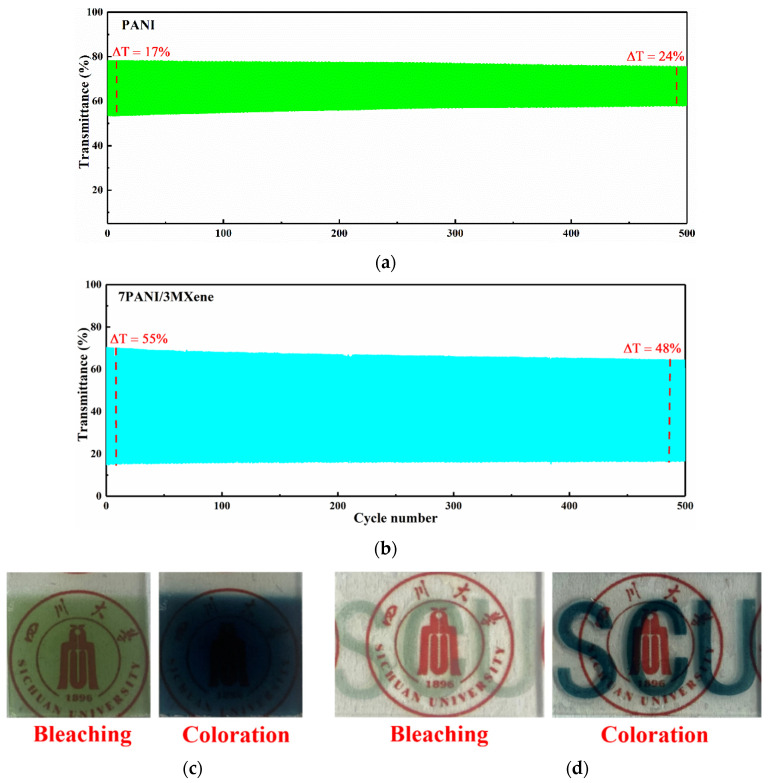
(**a**,**b**) Cycling stability testing of PANI and PANI/MXene films for 500 cycles at 700 nm; (**c**) photograph of PANI/MXene nanocomposite film at −0.4V (bleaching state) and +0.8V (coloration state); (**d**) photograph of the patterning electrochromic film based on PANI/MXene.

The cycling stability of the pure PANI and PANI/MXene films was measured by switching the potentials for 1–500 cycle switches between −0.4 V and +0.8 V, respectively, for 10 s. and recording the single-wavelength spectrophotometry at λ = 700 nm (Figure 7a,b). After 500 cycles, the optical contrast of the PANI/MXene film reduced from 55% to 48% (the ΔT retention is above 87%), while that of the pure PANI films decreased from 24% to 17% (the ΔT retention was only about 71%). It indicates that PANI/MXene films have good electrochemical stability as well as cycle life [24,47,57].

Photographs of the PANI/MXene film in the bleaching state and coloration state are displayed in Figure 7c. It showed as light yellow when the applied potential was −0.4 V (bleaching), and dark blue when it was at +0.8 V (coloration). In addition, a photograph of the patterned PANI/MXene electrochromic film is displayed in Figure 7d. It demonstrates that the self-assembly PANI/MXene not only can be easily fabricated into conventional large-area electrochromic devices by spray-coating technique, but also can be fabricated into patterned devices for electronic labels or displays.

**Figure 8:**
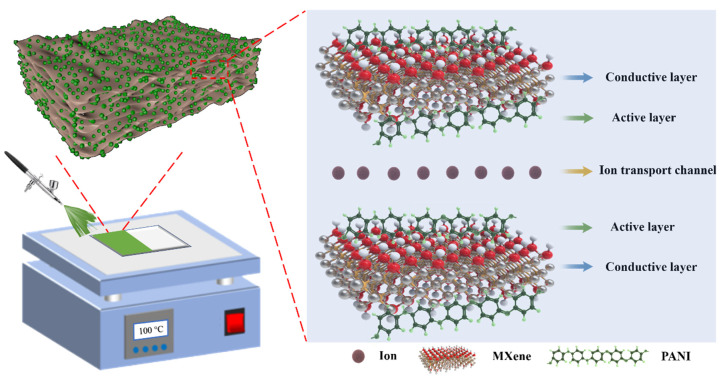
Schematic of the nanostructure of PANI/MXene for the electrochromic electrode.

The enhanced electrochromic behaviors of the PANI/MXene nanocomposite film compared with the pure PANI film could be attributed to the distinctive structure illustrated in Figure 8. The ion diffusion process has a great influence on the performance of electrochromic materials [48]. For the pure PANI films, the insertion/extraction of ions in charge transfer reaction are considerably difficult because of their dense structure (Figure 3b), where the ions are difficult to diffuse into the interior of the pure PANI film, which reduces the optical contrast and electrochemical response time. However, when the PANI nanoclusters were coupled with MXene nanosheets through the hydrogen bond interaction, the composite nanosheets with rough surfaces were obtained. By spraying process, the PANI/MXene nanocomposites formed a porous film (Figure 3c). As shown in Figure 8, this film with three-dimensional pore structure is conducive to the reversible electrochemical redox reaction. On the one hand, the holes in the film provide channels for ion transportation in the process of electrochemical doping and de-doping. On the other hand, the high conductivity of MXene (conductive layer) reduces the charge transfer resistance, which greatly improves the electrochemical activity of the film [58]. Furthermore, the PANI nanoclusters on the surface of MXene nanosheets fully exposed to the electrolytes also resulted in a fast electrochemical response. In a word, the synergistic effects between PANI and MXene facilitate the redox reaction kinetics and improve the electrochromic performance.

## 4. Conclusions

In summary, we successfully developed a facile and scalable strategy to prepare PANI/MXene nanocomposites, which can be easily fabricated into electrochromic film through a simple solution process technique. Well-defined PANI/MXene nanocomposites can be prepared through a self-assembly process in a mixed solution of PANI and few-layer MXene in which PANI nanoparticles are anchored on MXene nanosheets by a hydrogen-bonding interaction. Unlike the dense structure of pure PANI film, PANI/MXene film exhibits a porous structure. The composites showed an obvious synergetic effect of both materials leading to superior electrochromic properties, including large optical contrast (55% at λ = 700 nm), fast response time (1.3 s for coloration and 2.0 s for bleaching), and excellent cycle stability (after 500 cycles, the ΔT retention was above 87%). These excellent performances are attributed to the high conductivity of MXene and the formation of the porous composite film structure, which promote the charge transfer and ion migration efficiency. The robust composites also showed a good suspension stability and spray-coating processability, which facilitate the fabrication of large area films and patterning electrochromic devices. This research opens up a new approach to fabricate conductive polymer/MXene composite for application in the fields of electronic films and devices.

## Data Availability

Data sharing not applicable.

## Supplementary Materials

The following are available online at https://www.mdpi.com/article/10.3390/nano11112956/s1, Figure S1: Tyndall phenomenon of MXene diluted solution. Figure S2: (a,b) The SEM images of exfoliated Ti_3_C_2_T_x_ MXene. Figure S3: (a,b) SEM images of spray-coated films, (a) MXene; (b) PANI. Figure S4: (a) SEM image of PANI/MXene film. (b,c) TEM images of the PANI/MXene nanocomposite with different magnifications. Arrow points to the fold positions. Figure S5: Ti 2p spectra of PANI/MXene. Figure S6: Equivalent circuits that optimally fit with obtained Nyquist plots of PANI/MXene electrodes. Figure S7: Comparison of various UV-vis transmittance spectra of PANI/MXene film performed at different mass rates. Table S1: Contribution of oxygen groups resulting from the fitting of N 1s spectrum of MXene/PANI. Table S2: Electrical parameters for both of pure PANI and the PANI/MXene electrode evaluated from EIS test.
