# Supplementary material for: Self-Assembled Polyaniline/Ti3C2Tx Nanocomposites for High-Performance Electrochromic Films
Source: Nanomaterials (Basel). 2021 Nov 4;11(11):2956. doi: 10.3390/nano11112956 (PMC8623319; doi:10.3390/nano11112956)
Supplement: Supplementary file 1 [file nanomaterials-11-02956-s001.zip › nanomaterials-1449258-supplementary.pdf]

Supplementary

Self-Assembled Polyaniline/$Ti_3C_2T_x$ Nanocomposites for High-Performance Electrochromic Films

Tao Lin [1,2,3], Wenlong Liu [1,4,*], Bin Yan [2], Jing Li [4], Yi Lin [2], Yinghui Zhao [2], Zheng Shi [2] and Sheng Chen [2,*]

[1] School of Mechanical Engineering, Chengdu University, Chengdu 610106, China; lintao@stu.cdu.edu.cn
[2] College of Biomass Science and Engineering, Sichuan University, Chengdu 610065, China; yanbinscu@126.com (B.Y.); linasdn@126.com (Y.L.); yh18428367250@163.com (Y.Z.); drshiz1002@hotmail.com (Z.S.)
[3] Advanced Materials Deformation and Damage from Multi-Scale, Chengdu University, Chengdu 610106, China;
[4] College of Food and Biological Engineering, Chengdu University, Chengdu 610106, China; lijing@cdu.edu.cn
* Correspondence: liuwenlong_666@126.com (W.L.); chensheng@scu.edu.cn (S.C.)

Information Figures

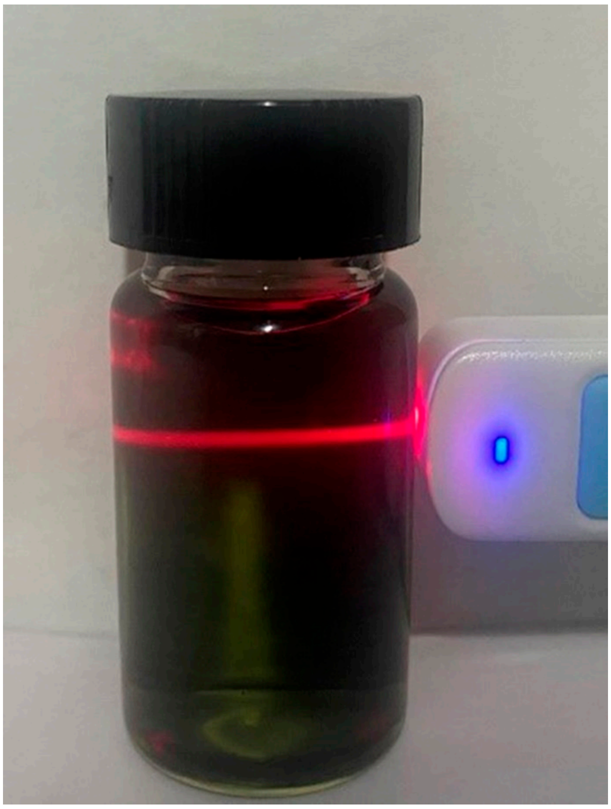

Figure S1. Tyndall phenomenon of MXene diluted solution.

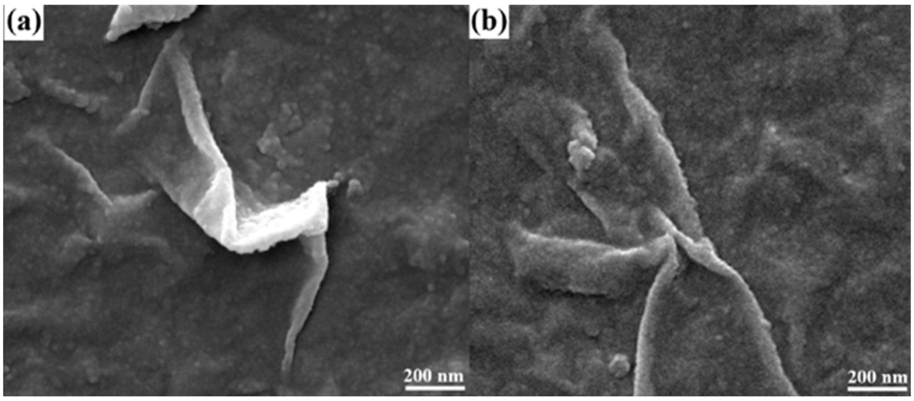

Figure S2. (a) and (b) The SEM images of exfoliated $Ti_3C_2T_x$ MXene.

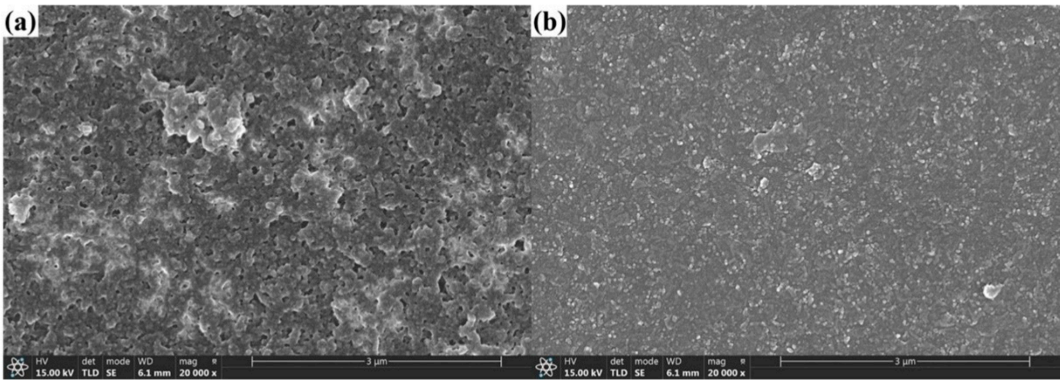

Figure S3. (a) and (b) SEM images of spray-coated films, (a) MXene; (b) PANI.

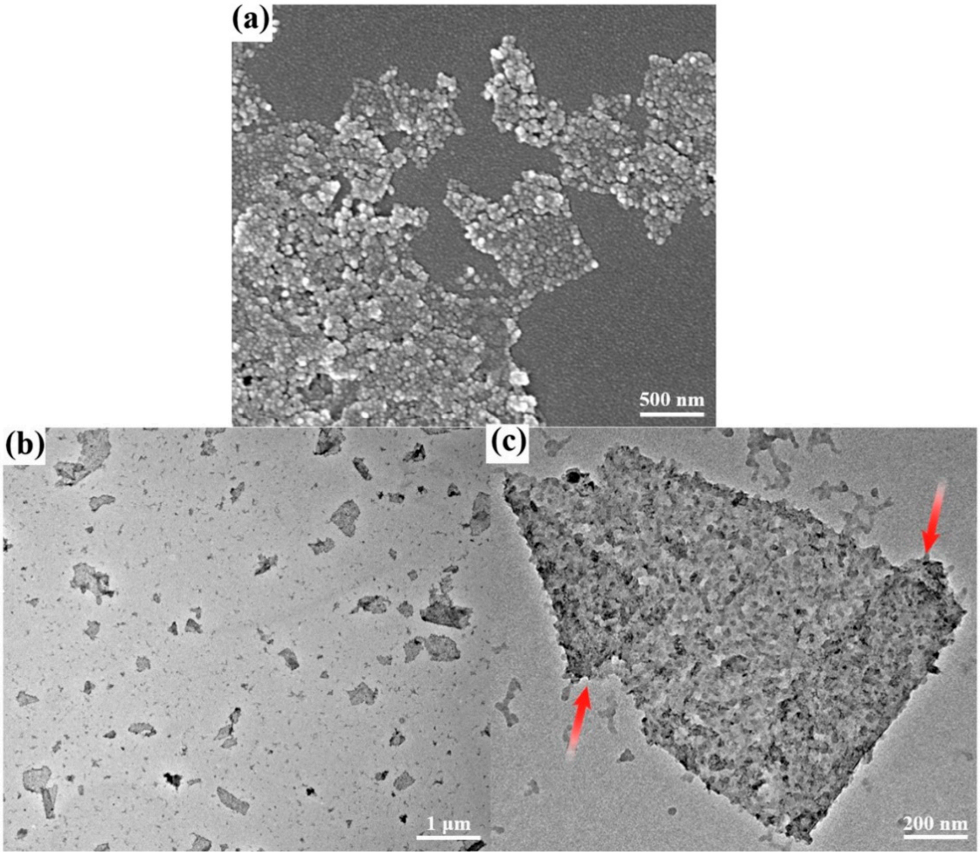

Figure S4. (a) SEM image of PANI/MXene film. (b) and (c) TEM images of the PANI/MXene nanocomposite with different magnifications. Arrow points to the fold positions.

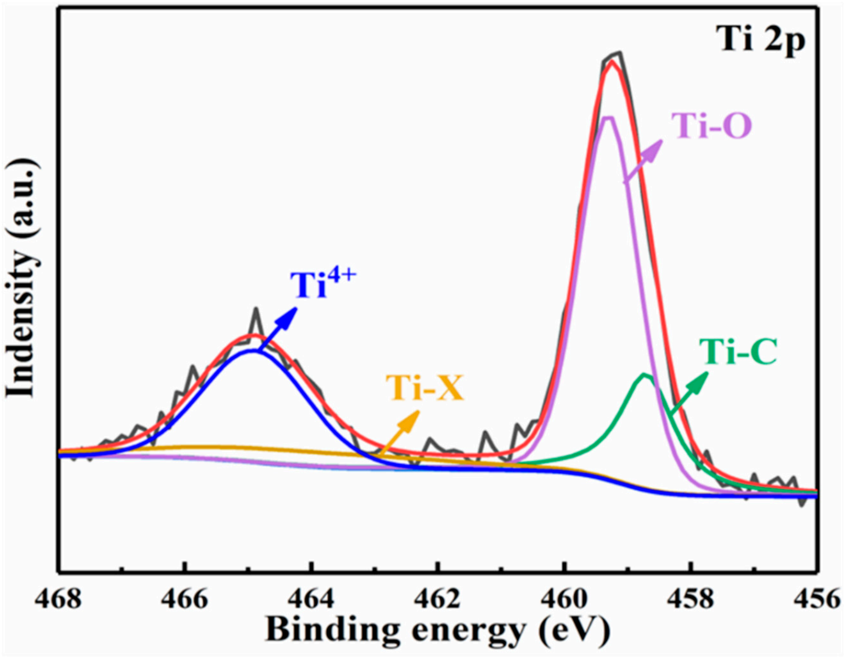

Figure S5. Ti 2p spectra of PANI/MXene.

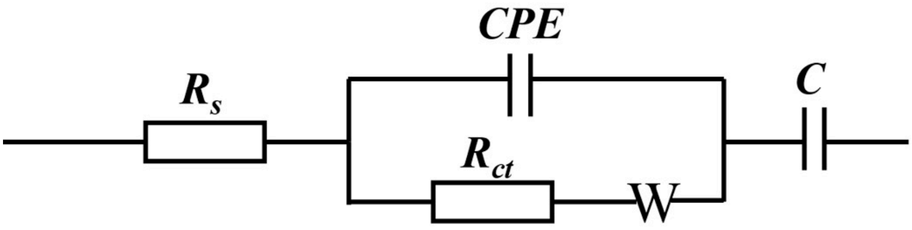

Figure S6. Equivalent circuits that optimally fit with obtained Nyquist plots of PANI/MXene electrodes.

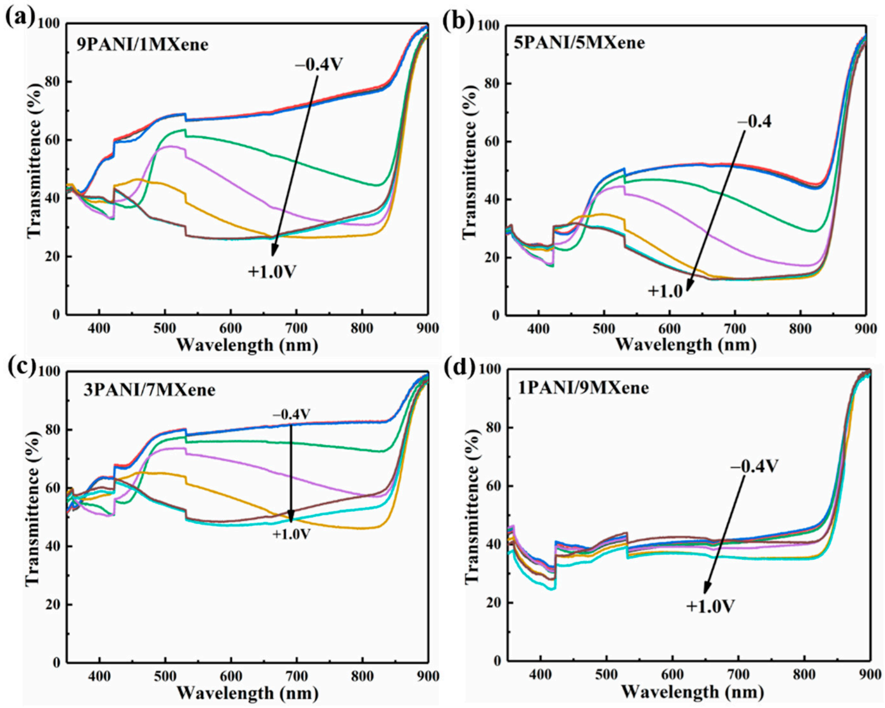

Figure S7. (a)-(d) Comparison of various UV-vis transmittance spectra of PANI/MXene film performed at different mass rates.

Table S1. Contribution of oxygen groups resulting from the fitting of N 1s spectrum of MXene/PANI.

Sample	Species	λ (nm)	Composition (%)	[=N—/N—]
PANI/MXene	=N-	398.9	32.68	0.72
	-NH-	399.7	45.38	
	N^{*+}	400.9	17.89	
	N^{+}	402.6	4.05	

Table S2. Electrical parameters for both of pure PANI and the PANI/MXene electrode evaluated from EIS test.

Sample	R_s (Ω)	R_{ct} (Ω)	W ($\Omega \cdot s^{-0.5}$)
Pure PANI	4.126	3.099	6.411
PANI/MXene	2.501	0.860	3.911
